# Comparison between tracheal ratio methods used by three observers at three occasions in English Bulldogs

**DOI:** 10.1186/s13028-014-0079-6

**Published:** 2014-12-16

**Authors:** Jessica Ingman, Veronica Näslund, Kerstin Hansson

**Affiliations:** University Animal Hospital, Swedish University of Agricultural Sciences, P.O. Box 7040, SE-750 07 Uppsala, Sweden; Alingsås Animal Hospital, Ridhusvägen 4, SE-441 93 Alingsås, Sweden; Department of Clinical Sciences, Faculty of Veterinary Medicine and Animal Science, Swedish University of Agricultural Sciences, P.O. Box 7054, SE-750 07 Uppsala, Sweden

**Keywords:** Brachycephalic, Congenital, Health screening, Index, Radiography, Tracheal hypoplasia

## Abstract

**Background:**

Tracheal hypoplasia is a congenital condition described in mainly brachycephalic breeds and is one component of the brachycephalic obstructive airway syndrome (BOAS). Two radiographic methods have been described to evaluate the dimensions of the tracheal diameter in dogs and to distinguish between hypoplastic and non-hypoplastic tracheas: the tracheal lumen diameter to thoracic inlet distance ratio (TD/TI) and the ratio between the thoracic tracheal luminal diameter and the width of the proximal third of the third rib (TT/3R). The purpose of this study was to compare these two published radiographic methods between observers, different measuring occasions and to investigate the effect on classification of dogs as having hypoplastic or non-hypoplastic tracheas using four previously published mean ratios as cut-offs (<0.11, <0.127 and <0.144 for the TD/TI and <2.0 for the TT/3R method).

Three observers evaluated right and left lateral recumbent radiographs from 56 adult English Bulldogs independently on three different occasions. TD/TI and TT/3R ratios were calculated and correlated between measuring occasions. Kappa, observed, positive, and negative agreements were calculated between observers and measuring occasions. Number of hypoplastic and non-hypoplastic dogs for each method and occasion was determined using <0.11, <0.127 and <0.144 as cut-offs for TD/TI and <2.0 for TT/3R.

**Results:**

Intraobserver agreement varied with kappa between 0.45-0.94 for the TD/TI and 0.20-0.86 for the TT/3R method. Interobserver kappa varied between 0.27-0.70 for the TD/TI method and between 0.05-0.57 for the TT/3R method. There was poor agreement in classifying English Bulldogs as tracheal hypoplastic or non-hypoplastic, depending on measuring method, cut-off value and observer.

**Conclusions:**

The diagnostic value of both the TD/TI and TT/3R methods with such poor agreement is questionable, and significantly impacts their reliability for both clinical evaluation of dogs and use in health screening programs.

## Background

Tracheal hypoplasia, a congenital condition predominantly of brachycephalic breeds, is one component of the brachycephalic obstructive airway syndrome (BOAS) and reported to be more common in English Bulldogs [1-4]. It is characterised by a markedly reduced tracheal lumen throughout the trachea [3,5]. The tracheal cartilages are small and rigid and their free ends closely appose or overlap, with a shortening of the dorsal elastic membrane and the trachealis muscle [2-4,6-8]. The diameter of a hypoplastic trachea does not vary during respiration [1,5]. Described clinical signs are similar as for BOAS, and include dyspnoea, stridor, coughing, gagging, choking, exercise intolerance and syncope [1]. Tracheal hypoplasia alone does not always cause clinical signs [1,9,10].

Several radiographic methods calculating different ratios used to evaluate the tracheal diameter have been published [1-3]. Suter *et al.* [3] calculated a ratio between the tracheal diameter and the width of the proximal third of the third rib. Whether this is luminal diameter or the entire tracheal diameter is not specified nor where the trachea was measured. Coyne and Fingland [1] modified the method by Suter *et al.* [3] by defining a ratio between the thoracic tracheal luminal diameter measured at the midpoint between the thoracic inlet and the carina (TT) and the width of the proximal third of the third rib (3R). The ratio of trachea to the third rib in a small number of normal dogs in Suter’s study was approximately 3:1 [3]. The study included six hypoplastic dogs with a ratio between 1:1 and 2:1. Either <2.0 or <3.0 are in later literature used as definition of tracheal hypoplasia [1,4,7,11-13].

A second method published by Harvey and Fink [2] calculated a tracheal lumen diameter to thoracic inlet distance ratio (TD/TI) and reported seven different mean ratios for Bulldogs that included ratios for all dogs, dogs with or without respiratory signs, dogs younger and older than one year and female and male dogs. The mean ratio for all 39 Bulldogs was 0.127 ± 0.038, regardless of age, sex or respiratory signs. The selective ratios varied between 0.113 ± 0.027 and 0.155 ± 0.038 with the lowest value for Bulldogs without respiratory signs. The most cited TD/TI ratio is 0.127. A later study by Coyne and Fingland [1] on tracheal hypoplasia included 13 Bulldogs without hypoplasia as controls with a mean TD/TI of 0.144 (SEM 0.009).

The two most widely referred methods today for evaluating the tracheal dimensions are the TD/TI ratio by Harvey and Fink [2] and the TT/3R ratio by Coyne and Fingland [1]. A mixture of different mean ratios is reported in subsequent literature referencing these early studies. Sometimes some of the ratios are erroneously referred to as established normal values [1,2,6,7,12-20].

Screening programmes, for tracheal hypoplasia, have been established or investigated in several countries [21-25]. Unfortunately published studies on the heritability of tracheal hypoplasia are lacking.

To the authors’ knowledge there is no study that evaluates the consistency in the resulting ratios using measurements of tracheal dimensions, rib width and thoracic inlet dimension or the agreement between the TD/TI and TT/3R methods. The purpose of this study was to investigate variability of these two published radiographic methods. The aim was to investigate if there would be any variation in radiographic classification of English Bulldogs as tracheal hypoplastic or non-hypoplastic, depending on measuring method, chosen cut-off ratio, observer, measuring occasion or recumbency. The hypothesis was that the subjectivity in selecting the measurement points would cause variation in calculated ratios with implication on the classification of dogs as tracheal hypoplastic or non-hypoplastic.

## Methods

The study was performed in collaboration with the Swedish Kennel Club and the Swedish Breed Club for English Bulldogs during 2007–2010. The breed club conveyed information about the study to all of its members and encouraged participation, which was voluntary. No pre-selection of dogs was done. Dogs could be radiographed at any veterinary clinic in the country and needed to be at least one year old. This was to simulate the situation in an actual health-screening programme. The owner brought a request provided by the breed club with written instructions to the imaging veterinarian. The study had ethical approval (no. C151/7), issued by Uppsala Ethical Review Committee on Animal Experiments, and the owner’s consent was obtained for each dog. The veterinarian was instructed to obtain thoracic radiographs during peak inspiration with the dog in right (RLR) and left lateral recumbency (LLR), the images marked with the recumbent side. The thoracic limbs should be pulled as cranially as possible in order to minimise superimposition with the cranial thorax. If possible the dog should not have been given any sedatives. If sedation or anaesthesia was necessary the substance, dose and any intubation needed to be recorded on the request. The request and radiographs were submitted to the Department of Clinical Sciences. Each dog was radiographed once. Radiographs were both analogue and in digital format, as either DICOM-files or printouts. Measurements on analogue images and printouts were performed with a ruler. Images in DICOM-format were viewed and measured in a commercial viewing system (GE Healthcare Centricity Radiology RA600 v8.0). In some digital images the precise mm-scaling was unknown and measurements were shown in units. This was not considered to affect the comparison of dogs since measurements were calculated into ratios.

Three observers evaluated all radiographs independently. The same radiographs of each dog, both RLR and LLR, were evaluated at three different occasions (in total nine measuring occasions) with a minimum of one day between the measuring occasions. The observers were: a last year veterinary student, observer A, a DipECVDI, observer B, and a resident in diagnostic imaging, observer C. For each radiograph at each occasion the tracheal lumen diameter at the thoracic inlet (TD) and the thoracic inlet distance (TI) was measured according to Harvey and Fink [2] and Coyne and Fingland [1], as well as the thoracic tracheal luminal diameter (TT) and the width of the proximal third of the third rib (3R) according to Coyne and Fingland [1]. TD/TI and TT/3R ratios were thereafter calculated for each measuring occasion. In order to mimic that various interpreters are using the methods during clinical work, and for screening in health programs, no consensus in how to measure the relevant diameters was agreed on between the observers prior to the study. It was up to each observer to interpret the method as described in the original articles by Coyne and Fingland [1] and Harvey and Fink [2].

### Statistical analyses

Cohen’s kappa (κ), total observed agreement (P_O_), positive (PA) and negative agreement (NA) were calculated between all nine measuring occasions (inter- and intra-agreement) for each RLR and LLR radiograph and each method using previously published cut-off ratios of <0.127 for the TD/TI and <2 for the TT/3R method as classification of tracheal hypoplasia. P_O_, PA and NA were calculated to discover and resolve possible paradoxes in the Cohen’s kappa calculations [26,27]. The kappa statistics were interpreted according to Landis and Koch [28]. Pearson’s correlation coefficients were calculated between each reader’s observation occasions. Number of dogs graded as hypoplastic was determined for each observer, each method, and in both RLR and LLR projections, with previously published cut-off values of <0.11, <0.127 and <0.144 for the TD/TI [1,2] and <2 for the TT/3R method [3]. Data were analysed with commercially available software (SAS/STAT software, version 9.3, Microsoft Excel 2011).

## Results

During the study period radiographs from 73 dogs were evaluated. Only dogs where all observers had performed all measurements in both RLR and LLR radiographs at all nine occasions were included. 17 dogs were consequently excluded due to incomplete measurements. Most common reasons for lack of measurement were that parts of the manubrium could not be visualized due to underexposure or poor positioning with superimposing forelimbs. 56 English Bulldogs were finally included. The dogs’ median age was 2.1 years (range 1–10.7 years) with 28 males and 28 females. Six dogs were sedated when radiographed, three with a combination of medetomidine (10-12 μg/kg) and butorphanol (0.1 mg/kg), one with a combination of dexmedetomidine (5 μg/kg) and butorphanol (0.1 mg/kg) and two with only dexmedetomidine (11-12 μg/kg). None were anaesthetized.

Inter- and intraobserver κ, P_O_, PA and NA for TD/TI and TT/3R methods (with cut-off ratios <0.127 and <2 respectively) are presented in Tables 1, 2, 3 and 4. Overall for the inter- and intra-agreements there was in most instances a higher P_O_, while the corresponding κ-values were lower, indicating paradoxical results due to low prevalence of positive cases (hypoplastic dogs) [26]. Regarding inter-agreement for the TD/TI method, κ varied between 0.27-0.70 and the PA between 0.35-0.79, which gave a variation in agreement from fair to substantial [28]. The inter-agreements for the TT/3R method varied from slight to moderate with a κ range of 0.05-0.57 and a PA of 0.12-0.63. The intra-agreement varied between moderate to almost perfect for the TD/TI method (κ 0.45-0.94 and PA 0.50-0.95) and between slight to almost perfect for the TT/3R method (κ 0.20-0.86 and PA 0.25-0.96). The κ, P_O_, PA and NA values were constantly lower for the TT/3R method for both inter- and intra-agreements. The inter- and intraobserver NA values were higher than the corresponding κ, P_0_ and PA values in all instances except for the intra-agreement values for reader A with the TT/3R method (Tables 1, 2, 3 and 4).

**Table 1:** **Inter-agreement kappa statistics for observers using the TD/TI method**

**Observer**	**Occasion 1**				**Occasion 2**				**Occasion 3**			
	**κ**	**P**_**O**_	**PA**	**NA**	**κ**	**P**_**O**_	**PA**	**NA**	**κ**	**P**_**O**_	**PA**	**NA**
**A vs. B**	0.66/0.52	0.88/0.66	0.74/0.59	0.92/0.93	0.49/0.62	0.82/0.88	0.58/0.79	0.87/0.92	0.67/0.62	0.88/0.88	0.74/0.70	0.92/0.92
**B vs. C**	0.56/0.70	0.88/0.95	0.63/0.73	0.92/0.97	0.45/0.52	0.89/0.89	0.50/0.57	0.94/0.94	0.69/0.52	0.89/0.89	0.67/0.57	0.94/0.94
**A vs. C**	0.45/0.36	0.82/0.86	0.55/0.43	0.89/0.92	0.32/0.27	0.79/0.80	0.40/0.35	0.87/0.88	0.45/0.45	0.80/0.84	0.56/0.53	0.87/0.90

Inter-agreement for the TD/TI method (with ratio <0.127 as definition of tracheal hypoplasia) for the different measurement occasions. Kappa (κ), observed agreement (P_O_), positive agreement (PA) and negative agreement (NA) values are presented for both right and left lateral recumbent radiographs (RLR/LLR). In total 56 RLR and 56 LLR radiographs.

**Table 2:** **Inter-agreement kappa statistics for observers using the TT/3R method**

**Observer**	**Occasion 1**				**Occasion 2**				**Occasion 3**			
	**κ**	**P**_**O**_	**PA**	**NA**	**κ**	**P**_**O**_	**PA**	**NA**	**κ**	**P**_**O**_	**PA**	**NA**
**A vs. B**	0.13/0.17	0.48/0.54	0.36/0.32	0.57/0.65	0.07/0.08	0.36/0.41	0.22/0.20	0.45/0.54	0.06/0.10	0.34/0.41	0.21/0.27	0.43/0.51
**B vs. C**	0.38/0.21	0.84/0.89	0.47/0.25	0.91/0.94	0.26/0.56	0.86/0.93	0.33/0.60	0.92/0.96	0.57/0.39	0.89/0.88	0.63/0.46	0.94/0.93
**A vs. C**	0.11/0.05	0.46/0.46	0.32/0.12	0.56/0.62	0.10/0.12	0.39/0.45	0.29/0.28	0.47/0.55	0.15/0.12	0.45/0.43	0.42/0.30	0.47/0.52

Inter-agreement for the TT/R3 method (with ratio <2 as definition of tracheal hypoplasia) for the different measurement occasions. Kappa (κ), observed agreement (P_O_), positive agreement (PA) and negative agreement (NA) values are presented for both right and left lateral recumbent radiographs (RLR/LLR). In total 56 RLR and 56 LLR radiographs.

**Table 3:** **Intra-agreement kappa statistics for measurement occasion with the TD/TI method**

**Occasion**	**Observer A**				**Observer B**				**Observer C**			
	**κ**	**P**_**O**_	**PA**	**NA**	**κ**	**P**_**O**_	**PA**	**NA**	**κ**	**P**_**O**_	**PA**	**NA**
**1 vs. 2**	0.69/0.62	0.88/0.88	0.77/0.70	0.91/0.92	0.51/0.79	0.86/0.95	0.60/0.82	0.91/0.97	0.50/0.73	0.91/0.96	0.55/0.75	0.95/0.98
**2 vs. 3**	0.87/0.85	0.95/0.95	0.91/0.89	0.96/0.96	0.87/0.94	0.96/0.98	0.89/0.95	0.98/0.99	0.45/0.88	0.89/0.98	0.50/0.89	0.94/0.99
**1 vs. 3**	0.65/0.79	0.86/0.93	0.75/0.83	0.90/0.95	0.66/0.85	0.89/0.96	0.73/0.88	0.93/0.98	0.78/0.64	0.95/0.95	0.80/0.67	0.97/0.97

Intra-agreement for the TD/TI method (with ratio <0.127 as definition of tracheal hypoplasia) for the different measurement occasions. Kappa (κ), observed agreement (P_O_), positive agreement (PA) and negative agreement (NA) values are presented for both right and left lateral recumbent radiographs (RLR/LLR). In total 56 RLR and 56 LLR radiographs.

**Table 4:** **Intra-agreement kappa statistics for measurement occasion with the TT/3R method**

**Occasion**	**Observer A**				**Observer B**				**Observer C**			
	**κ**	**P**_**O**_	**PA**	**NA**	**κ**	**P**_**O**_	**PA**	**NA**	**κ**	**P**_**O**_	**PA**	**NA**
**1 vs. 2**	0.63/0.74	0.84/0.88	0.88/0.90	0.74/0.84	0.68/0.78	0.93/0.96	0.71/0.80	0.96/0.98	0.62/0.20	0.91/0.89	0.67/0.25	0.95/0.94
**2 vs. 3**	0.86/0.75	0.95/0.89	0.96/0.92	0.90/0.83	0.78/0.78	0.96/0.96	0.80/0.80	0.98/0.98	0.74/0.39	0.93/0.88	0.78/0.46	0.96/0.93
**1 vs. 3**	0.67/0.74	0.86/0.88	0.95/0.90	0.76/0.83	0.52/0.63	0.89/0.93	0.57/0.67	0.94/0.96	0.68/0.41	0.91/0.91	0.74/0.44	0.95/0.95

Intra-agreement for the TT/3R method (with ratio <2 as definition of tracheal hypoplasia) for the different measurement occasions. Kappa (κ), observed agreement (P_O_), positive agreement (PA) and negative agreement (NA) values are presented for both right and left lateral recumbent radiographs (RLR/LLR). In total 56 RLR and 56 LLR radiographs.

**Figure 1:**
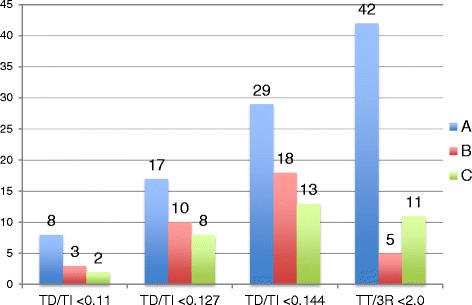
**Bar graph with number of dogs classified as having tracheal hypoplasia by three observers in RLR radiographs.** Number of dogs classified as tracheal hypoplastic by readers A, B and C at the 3rd measuring occasion in RLR projections, using <0.11, <0.127 and <0.144 as cut-off ratios for the TD/TI and <2 for the TT/3R methods.

**Figure 2:**
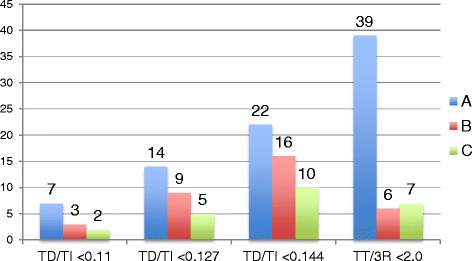
**Bar graph with number of dogs classified as having tracheal hypoplasia by three observers in LLR radiographs.** Number of dogs classified as tracheal hypoplastic by readers A, B and C at the 3rd measuring occasion in LLR projections, using <0.11, <0.127 and <0.144 as cut-off ratios for the TD/TI and <2 for the TT/3R methods.

When comparing Pearson’s correlation coefficients between individual observers’ measuring occasions for the TD/TI method, the highest correlation for all three observers was seen between the 2^nd^ and 3^rd^ occasions (range 0.96-0.97). The correlations for the TT/R3 method were also highest between the 2^nd^ and 3^rd^ occasions for observers A and C (0.92 and 0.83 respectively), but not for observer B who had the highest correlation between the 1^st^ and 2^nd^ occasions. However observer B had more even correlations for the TT/3R method with 0.81 between the 1^st^ and 2^nd^ and 0.78 between the 2^nd^ and 3^rd^ occasions. The correlations were higher for the TD/TI method than for the TT/3R method. The correlations were interpreted as indicative of some degree of learning and therefore ratios from the 3^rd^ occasion were used in analysing number of dogs classified as tracheal hypoplastic by using ratios <0.11, <0.127 and <0.144 as cut-offs for the TD/TI method and <2 for the TT/3R method. Number of dogs classified as hypoplastic by each reader in RLR and LLR radiographs and for the different cut-offs at occasion three are presented in Figures 1 and 2.

Reporting mean ratios was not the purpose of the study, however when calculating correlations also mean TD/TI ratios were obtained for all nine measuring occasions and for both RLR and LLR radiographs. The mean ratios for both the TD/TI and TT/3R methods were at all occasions lower in RLR projections.

Despite method and cut-off, observer A classified more dogs as hypoplastic in comparison with the two other observers.

When using <0.127 for TD/TI in combination with <2 for TT/3R as cut-off for tracheal hypoplasia classification, not one single dog was classified as hypoplastic by both methods, and all observers at every measuring occasion. 10 dogs were classified as non-hypoplastic and the remaining 46 dogs had a varying classification.

## Discussion

There does not exist any radiological gold standard for diagnosing tracheal hypoplasia. Therefore true prevalence is unknown. Instead the observed marginal total values in the concordance tables used in Cohen’s kappa calculations become the surrogates for prevalence. It is a known paradox in kappa-statistics that with symmetrical imbalanced marginal total values, κ will be reduced [26,27,29]. This was seen in the present study and therefore also PA was calculated and presented. Even though the PA were higher than the κ-values there was still a vast inter- and intraobserver variation shown by the range of κ and PA values (Tables 1, 2, 3 and 4). The intra-agreement was better than the inter-agreement, which would be expected. Both inter- and intra-agreement was worse for the TT/3R method most likely due to difficulties in defining the same measurement points when re-measuring the diameters. Suter *et al.* [3] does not specify where the tracheal diameter should be measured and Coyne and Fingland [1] describes for the TT/3R method that the thoracic tracheal luminal diameter should be measured at the midpoint between the thoracic inlet and the carina, which is rather subjective since the TT/3R method does not describe how to define the point of the thoracic inlet [1,3].

NA was always within the “almost perfect range” for the TD/TI method regardless of reader or occasion. However due to the expected higher prevalence of non-hypoplastic dogs the NA is also expected to be higher. The interobserver NA for the TT/3R method were lower, probably owing to observers B and C generally classifying fewer dogs as hypoplastic. Reader A consistently classified more dogs as hypoplastic using the TT/3R method (Figures 1 and 2). When investigating the individual measurements before calculating the ratios it could be seen that reader A consistently measured a slightly lower TT in combination with a higher 3R in comparison with the others. A lower TT in combination with a higher 3R will result in a lower ratio and minor differences in measurements resulted in major differences in the ratios. As an example when comparing RLR and LLR projections of one of the dogs at one reading occasion the TT/3R ratio in the RLR projection was 6 mm/4 mm = 1.5 and in the LLR projection 7 mm/3.5 mm = 2.0. A 1 mm increase in TT simultaneously as a 0.5 mm decrease in 3R resulted in a 0.5 discrepancy in the ratios. The same phenomenon was seen when a TD/TI ratio in RLR projections was measured as 7 mm/53 mm = 0.123 and 8 mm/52 mm = 0.154 in the corresponding LLR projection. Thus a 1 mm increase in TD and a 1 mm decrease in TI gave a 0.031 difference in the ratios.

No consensus in how to measure was agreed on between the observers prior to the study. The purpose with this was to simulate the situation for veterinary surgeons in daily practice, and for scrutinizers at breeding organizations in different countries, where each veterinarian or organization have to interpret the published methods by themselves without availability to an international consensus agreement. Agreeing on a consensus interpretation of the methods might have influenced the interobserver results in this study to a better agreement, however the inter-agreement would still not be expected to be higher than the intra-agreement.

The difference in radiological experience likely contributes partly to the varying agreements in this study, since one observer (A) was a last year veterinary student and one (C) a resident in diagnostic imaging. However, reader B (DipECVDI) with considerable experience in radiographic interpretation also had variable intra-agreement, with the lowest κ of 0.50 and lowest PA of 0.55 for the TD/TI method and κ as low as 0.20 and PA 0.25 for the TT/3R method.

Mean ratios for both the TD/TI and TT/3R methods were at all occasions lower in RLR projections. Previously published studies do not report which projections were used [1-3,6]. In dogs the trachea normally deviates slightly to the right at the thoracic inlet and in the cranial mediastinum and even more so in brachycephalic breeds*.* Measuring the trachea in this region could potentially give higher tracheal diameter measurements in LLR projections due to geometric magnification, and could explain the higher mean ratios calculated in these projections.

Many authors advocate ensuring the radiographs are true laterals when measuring. However due to the cone-shaped divergence of the x-ray beam this will never be the case for the entire radiograph. Since centering of the beam is done over the mid-thorax, there will be different degree of distortion of position and magnification of ribs in the periphery, as well as magnification of ribs further away from the film/imaging plate. Both Harvey and Fink [2] and Coyne and Fingland [1] mention the midpoint of the most cranial or first rib as a landmark when measuring the thoracic inlet with the TD/TI method. In this study the positioning of the most cranial rib in relation to the vertebrae varied. In some instances the most cranial rib superimposed with the seventh cervical vertebra, sometimes with the cranial endplate or body of the first thoracic vertebra. Any rotation in combination with congenitally malformed vertebrae, magnification and distortion of position sometimes made it difficult to assess which ribs was the third pair of ribs and sometimes only one of the third ribs could be confidently identified. Superimposition of ribs also obscured their margins. The TT/3R method by Coyne and Fingland [1] or Suter *et al.* [3] does not describe which rib, dependent or non-dependent, should be measured or if the *cartilago costalis* should be included when choosing the level of measurement. From the illustration in the study by Coyne and Fingland [1] however they seem to have only measured the *os costale.* The normal shape of the *os costale* is caudolaterally convex. Due to varying convexity it can appear shorter or longer in a two dimensional radiograph and much of the dorsal part of the ribs superimpose with the spine in many radiographs.

Several other variations were noted. The dogs had a variable conformation of the thorax with differing degree and amount of congenitally malformed vertebrae and some had flatter chests. In dogs with flatter chests the manubrium had a relatively more cranial positioning in relation to the first thoracic vertebra, which could potentially give a relatively higher TI measurement. Some dogs had varying degrees of flaccid dorsal tracheal membrane, making the true dorsal margin of the trachea appear indistinct and could contribute to varying measurements. The tracheal diameter can also vary between inspiration and expiration. One study showed that tracheal cross-sectional area changed by up to 20% in the thoracic-inlet region and 18.6% in the thoracic region. The phenomenon appeared to be directly related to changes in the cross-sectional shape of the trachea, with the largest percentage change in tracheal dimensions between inspiration and expiration seen in the tracheal height [30].

Considering that 1 mm differences in measurements can substantially affect the calculated ratios, any of the variations discussed above can potentially influence the tracheal hypoplasia classification of individual dogs.

## Conclusions

This study shows a poor agreement in classification of English Bulldogs as tracheal hypoplastic or non-hypoplastic, depending on measuring method, cut-off value and observer. The diagnostic value of both the TD/TI and TT/3R methods with such poor inter- and intra-agreement as well as poor agreement between methods must be questioned. This will have a significant impact on both clinical evaluation of dogs and on health screening programs for tracheal hypoplasia. Neither the TD/TI nor the TT/3R methods are useful since there is a lack of normal values, a gold standard and numerous mean values exist for the described methods. The heritability of tracheal hypoplasia is also unknown.

## Abbreviations

TD/TI: Tracheal luminal diameter to thoracic inlet distance ratio
TT/3R: Ratio between thoracic tracheal luminal diameter and width of the proximal third of the third rib
κ: kappa
P_O_: Observed agreement
PA: Positive agreement
NA: Negative agreement
RLR: Right lateral recumbent
LLR: Left lateral recumbent
